# Maternal mortality ratio in China from 1990 to 2019: trends, causes and correlations

**DOI:** 10.1186/s12889-021-11557-3

**Published:** 2021-08-11

**Authors:** Lu Chen, Penghui Feng, Lance Shaver, Zengwu Wang

**Affiliations:** 1 Division of Prevention and Community Health, Fuwai Hospital, Peking Union Medical College & Chinese Academy of Medical Sciences, no. 15 (Lin), Fengcunxili, Mentougou District, Beijing, 102308 China; 2 Department of Obstetrics and Gynecology, Peking Union Medical College Hospital, Chinese Academy of Medical Sciences & Peking Union Medical College, Beijing, 100730 China; 3 Faculty of Medicine, University of British Columbia, Vancouver, BC V6T 1Z4 Canada

**Keywords:** Maternal mortality ratio, Maternal death, Health financing composition, Obstetric hemorrhage, Hospital delivery rate

## Abstract

**Background:**

Maternal mortality ratio is an important indicator to evaluate the health status in developing countries. Previous studies on maternal mortality ratio in China were limited to certain areas or short periods of time, and there was a lack of research on correlations with public health funding. This study aimed to assess the trends in the maternal mortality ratio, the causes of maternal death, and the correlations between maternal mortality ratio and total health financing composition in China from 1990 to 2019.

**Methods:**

Data in this longitudinal study were collected from the China Health Statistics Yearbooks (1991–2020) and China Statistical Yearbook 2020. Linear regression analysis was used to assess the trends in the maternal mortality ratio in China. Pearson correlation analysis was used to assess the correlations between national maternal mortality ratio and total health financing composition.

**Results:**

The yearly trends of the national, rural and urban maternal mortality ratio were − 2.290 (*p* < 0.01), − 3.167 (*p* < 0.01), and − 0.901 (*p* < 0.01), respectively. The gap in maternal mortality ratio between urban and rural areas has narrowed. Obstetric hemorrhage was the leading cause of maternal death. The mortalities ratios for the main causes of maternal death all decreased in China from 1990 to 2019. The hospital delivery rate in China increased, with almost all pregnant women giving birth in hospitals in 2019. Government health expenditure as a proportion of total health expenditure was negatively correlated with the maternal mortality ratio (*r* = − 0.667, *p* < 0.01), and out-of-pocket health expenditure as a proportion of total health expenditure was positively correlated with the maternal mortality ratio (*r* = 0.516, *p* < 0.01).

**Conclusion:**

China has made remarkable progress in improving maternal survival, especially in rural areas. The maternal mortality ratio in China showed a downward trend over time. To further reduce the maternal mortality ratio, China should take effective measures to prevent obstetric hemorrhage, increase the quality of obstetric care, improve the efficiency and fairness of the government health funding, reduce income inequality, and strengthen the medical security system.

## Background

Maternal death remains a major global concern. Reducing maternal mortality ratio (MMR) is a priority goal on the international agenda [1]. Maternal mortality ratio is an important health indicator that can be used in making comparisons across different time periods and geographic regions [2]. Millennium Development Goal 5 was launched by members of the United Nations (UN)—its aim was to reduce the MMR by three-quarters between 1990 and 2015 [3–5]. During this period, China achieved the goal set by Millennium Development Goal 5 [6]. In 2015, all UN member states set the Sustainable Development Goal which aims at reduce the MMR to 70.0 per 100,000 live births by 2030 [7]. In 2016, the Chinese government proposed the Healthy China 2030 plan, which aimed to reduce the MMR to 18.0/100,000 births by 2020 and 12.0/100,000 births by 2030 [8]. This plan puts forward higher requirements for maternal health than proposed by the UN.

In the past three decades, China had launched several public health programs that are beneficial to maternal health, such as the Basic Public Health Service Equalization, Maternal Mortality Reduction and Neonatal Tetanus Elimination Program, Urban Employees Basic Medical Insurance, Five Strategies for Maternal and Newborn Safety, New Cooperative Medical Scheme, and Urban Residents Basic Medical Insurance [9–12]. After China implemented the Two-Child policy in 2013, the number of live births and the proportion of high-risk pregnancies increased, making it challenging to achieve the MMR target in the Healthy China 2030 plan [13, 14]. The main causes of maternal death include obstetric hemorrhage, puerperal infection, amniotic fluid embolism, pregnancy-induced hypertension, liver disease, and heart disease, which are mostly preventable [15]. Hospital delivery means that pregnant women give birth in hospital, which is an effective strategy to prevent maternal and infant deaths [16].

The total health expenditure (THE) is the total amount of money in currency consumed by the whole society for health services in a region or country in a certain period [17]. It includes government health expenditure (GHE), social health expenditure (SHE) and out-of-pocket health expenditure (OOPHE). THE can reflect the relationship between health policies and economic development, and provide information for the government to formulate health strategies. SHE is mainly composed of two parts: basic medical insurance fund and social capital investment paid by units and individuals [17]. The proportion of OOPHE is positively correlated with underdevelopment and social injustice [18, 19]. Health status is not only related to the amount of THE, but also the structure among the three components [20].

Although several studies have investigated the MMR in China, these studies were limited to certain areas [21–23] or short periods of time [24–26]. Further, there was a lack of research on the correlation between MMR and health financing composition. In this study, we aimed to assess the trends of MMR, the main causes of maternal death, and the correlations between MMR and total health financing composition in China from 1990 to 2019.

## Methods

### Definitions

Maternal deaths are defined as deaths of women who are pregnant or are within 42 days of termination of pregnancy, irrespective of the duration and site of the pregnancy, from any cause related to or aggravated by the pregnancy or its management, but not from accidental or incidental causes [27]. MMR is defined as the number of maternal deaths per 100,000 live births.

### Data sources

We conducted a longitudinal study. Data on the MMR, main causes of maternal death, hospital delivery rate, THE, CHE, SHE, and OOPHE from 1990 to 2019 were collected from the China Health Statistics Yearbooks (1991–2020). Data on per capita annual income from 1990 to 2019 came from the China Statistical Yearbook 2020.

To monitor the maternal deaths in China, National Maternal Mortality Surveillance System (NMMSS) was set up by the Chinese government in 1989. The sampling unit of NMMSS is at the county (district) level [9, 28]. In total, 336 surveillance spots (126 in urban areas and 210 in rural areas) across 31 provinces in mainland China were selected to record changes in MMR and the main causes of maternal death. The NMMSS has rigorous quality control mechanisms, including data audits, regular supervision, and standardization of data collection methods. Data on live births and maternal death are collected annually by trained officials and verified by government administrators before being included in the China Health Statistics Yearbooks [24].

### Statistical analysis

We analyzed the time series data using linear regression analysis to assess the trends in the MMR in China from 1990 to 2019 [9]. Pearson correlation analysis was used to assess the associations between the national MMR and China’s total health financing composition. All analyses in this study were conducted using Statistical Product and Service Solutions version 19, and a two-sided *p* value of < 0.01 was considered significant.

## Results

**Fig. 1:**
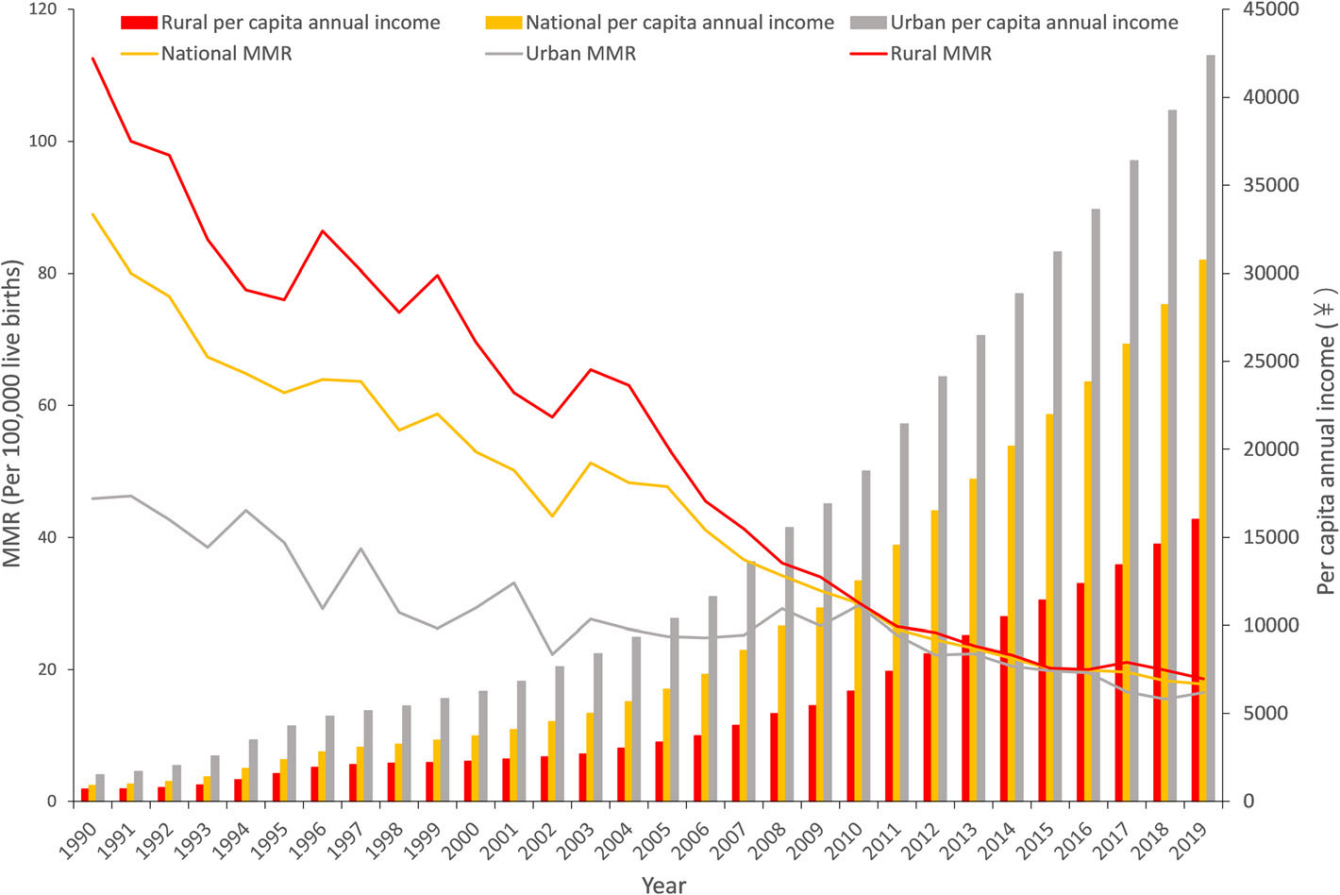
Maternal mortality ratio and per capita annual income in China from 1990 to 2019

The national MMR decreased by 80.0% from 88.9 per 100,000 live births in 1990 to 17.8 per 100,000 live births in 2019 (Fig. 1). Between 1990 and 2019, the rural MMR declined from 112.5 per 1000 livebirths to 18.6 per 1000 livebirths—a drop of 83.5%. The urban MMR decreased by 64.1% from 45.9 per 100,000 live births in 1990 to 16.5 per 100,000 live births in 2019. The MMR in rural areas was approximately twice (112.5 / 45.9 = 2.4) that in urban areas in 1990, and the difference between them (18.6 / 16.5 = 1.1) was relatively small in 2019. The per capita annual income of urban residents was 2.2 times (1510 / 686 = 2.2) that of rural residents in 1990, and it was 2.6 times (42,359 / 16,021 = 2.6) that of rural residents in 2019.

The results of the linear regression analysis of the national MMR showed that the coefficient was − 2.290 (95%CI: − 2.461, − 0.79; *p* < 0.01) and the intercept was 4635.412 (95%CI: 4292.435, 4978.389; *p* < 0.01). The trend of the yearly national MMR was − 2.290, which indicates a yearly decline of 2.290 maternal deaths per 100,000 livebirths. The results of linear regression analysis of the rural MMR showed that the coefficient was − 3.167 (95%CI: − 3.437, − 2.898; *p* < 0.01) and the intercept was 6403.053 (95%CI: 5863.390, 6942.717; *p* < 0.01), which indicates a yearly decline of 3.167 rural maternal deaths per 100,000 livebirths. The results of linear regression analysis of the urban MMR showed that the coefficient was − 0.901 (95%CI: − 1.070, − 0.731; *p* < 0.01) and the intercept was 1834.014 (95%CI: 1494.680, 2173.348; *p* < 0.01), which indicates a yearly decline of 0.901 urban maternal deaths per 100,000 livebirths.

**Fig. 2:**
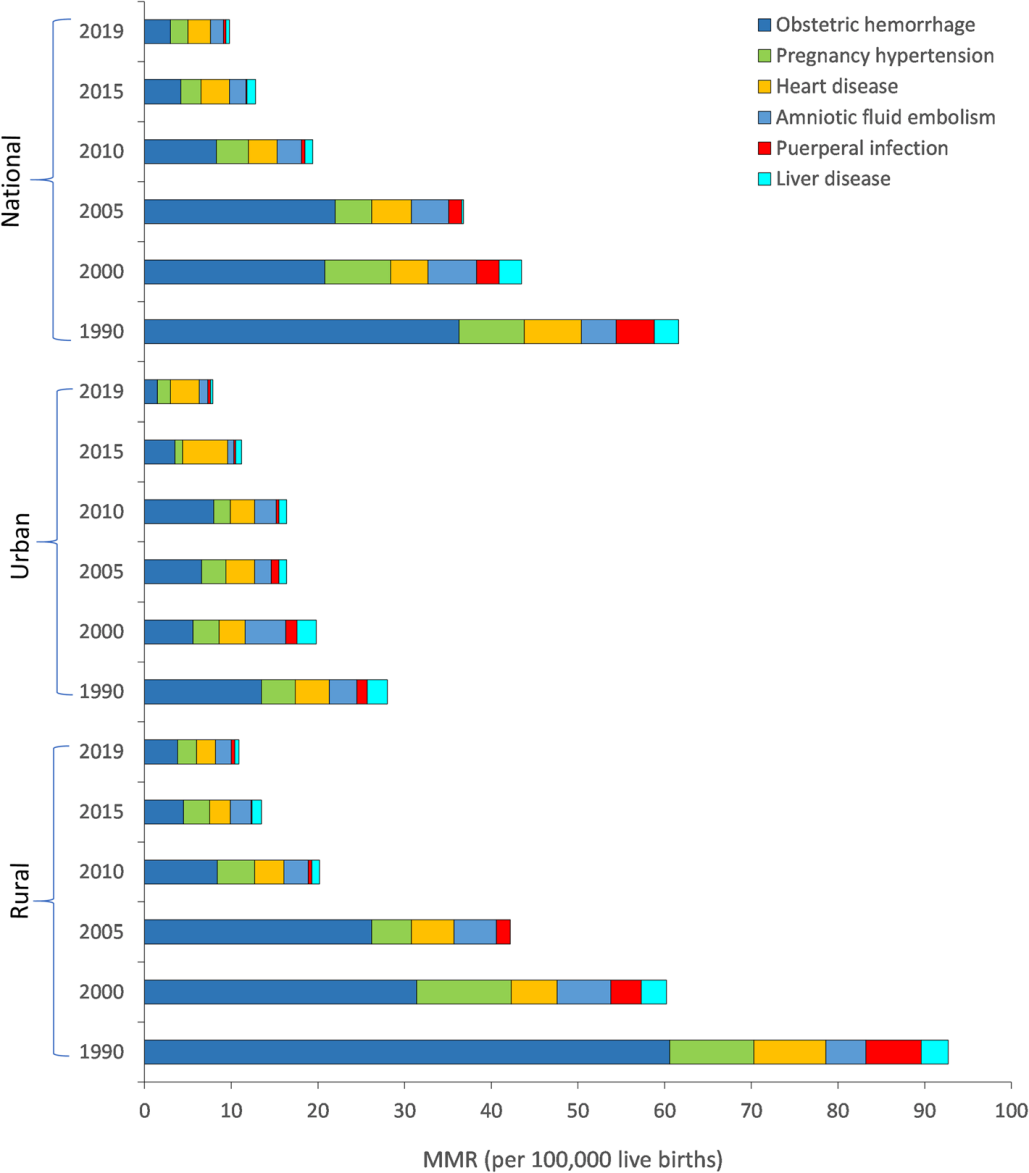
Maternal mortality ratio from main causes of pregnancy-related death in China, 1990–2019

**Fig. 3:**
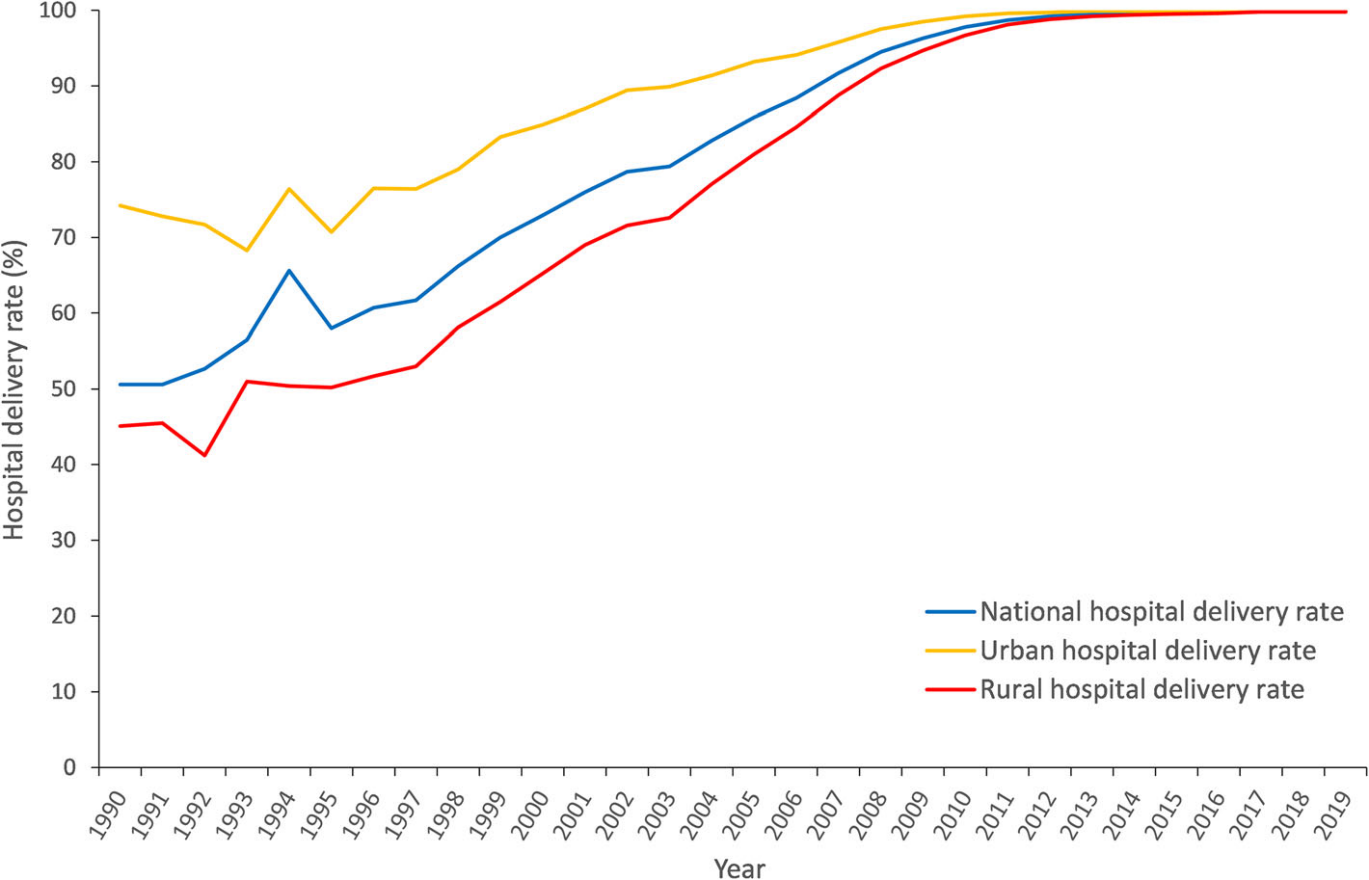
Hospital delivery rate in China from 1990 to 2019

The national MMR caused by puerperal infection dropped by 93.2% from 4.4 per 100,000 live births in 1990 to 0.3 per 100,000 live births in 2019 (Fig. 2); the national MMR caused by obstetric hemorrhage dropped by 91.7% from 36.3 per 100,000 live births in 1990 to 3.0 per 100,000 live births in 2019; the national MMR caused by liver disease dropped by 85.7% from 2.8 (1990) to 0.4 (2019). From 1990 to 2019, the national MMR caused by pregnancy-related hypertension, amniotic fluid embolism, and heart disease dropped by 73.3, 62.5, and 60.6%, respectively. The mortalities for the main causes of maternal death all decreased from 1990 to 2019. The national MMR caused by obstetric hemorrhage was 3.0 per 100,000 live births in 2019, which is the leading cause of maternal death nationwide. The leading cause of maternal death in urban areas was heart disease (3.3 per 100,000 live births) in 2019, while that in rural areas was obstetric hemorrhage (3.8 per 100,000 live births). The hospital delivery rate in China increased, with almost all pregnant women giving birth in hospitals in 2019 (Fig. 3). The hospital delivery rate in urban areas was 1.65 times (74.2 / 45.1 = 1.65) that in rural areas in 1990, and it was almost equal in urban and rural areas in 2019. The gap in hospital delivery rate between urban and rural areas declined gradually from 1990 to 2019.

The absolute number of THE, GHE, SHE and OOPHE increased in China from 1990 to 2019 (Table 1). The proportion of GHE and SHE in THE increased by 9.2 and 12.9% from 1990 to 2019, respectively. The proportion of OOPHE in THE decreased by 20.6% from 35.73% in 1990 to 28.36% in 2019. Pearson correlation analysis results showed that national MMR was negatively correlated with the absolute number of THE, GHE, SHE and OOPHE (*p* < 0.01). National MMR was negatively correlated with the proportion of THE in GDP (*r* = − 0.848, *p* < 0.01), and the proportion of GHE in THE (*r* = − 0.667, *p* < 0.01). There was a positive correlation between the national MMR and proportion of OOPHE in THE (*r* = 0.516, *p* < 0.01). National MMR was not significantly correlated with the proportion of SHE in THE (*r* = − 0.288, *p* = 0.122).

**Table 1:** Pearson correlations between national maternal mortality ratio and total health financing composition

	THE		GHE		SHE		OOPHE	
	Absolute number (billion Yuan)	Proportion in GDP (%)	Absolute number (billion Yuan)	Proportion in THE (%)	Absolute number (billion Yuan)	Proportion in THE (%)	Absolute number (billion Yuan)	Proportion in THE (%)
1990	74.74	3.96	18.73	25.06	29.31	39.22	26.70	35.73
1991	89.35	4.06	20.41	22.84	35.44	39.67	33.50	37.50
1992	109.69	4.03	22.86	20.84	43.16	39.34	43.67	39.81
1993	137.78	3.86	27.21	19.75	52.48	38.09	58.10	42.17
1994	176.12	3.62	34.23	19.43	64.49	36.62	77.41	43.95
1995	215.51	3.51	38.73	17.97	76.78	35.63	100.00	46.4
1996	270.94	3.77	46.16	17.04	87.57	32.32	137.22	50.64
1997	319.67	4.01	52.36	16.38	98.41	30.78	168.91	52.84
1998	367.87	4.32	59.01	16.04	107.10	29.11	201.76	54.85
1999	404.75	4.47	64.10	15.84	114.60	28.31	226.06	55.85
2000	458.66	4.57	70.95	15.47	117.19	25.55	270.52	58.98
2001	502.59	4.53	80.06	15.93	121.14	24.10	301.39	59.97
2002	579.00	4.76	90.85	15.69	153.94	26.59	334.21	57.72
2003	658.41	4.79	111.69	16.96	178.85	27.16	367.87	55.87
2004	759.03	4.69	129.36	17.04	222.54	29.32	407.14	53.64
2005	865.99	4.62	155.25	17.93	258.64	29.87	452.10	52.21
2006	984.33	4.49	177.89	18.07	321.09	32.62	485.36	49.31
2007	1157.40	4.28	258.16	22.31	389.37	33.64	509.87	44.05
2008	1453.54	4.55	359.39	24.73	506.56	34.85	587.59	40.42
2009	1754.19	5.03	481.63	27.46	615.45	35.08	657.12	37.46
2010	1998.04	4.84	573.25	28.69	719.66	36.02	705.13	35.29
2011	2434.59	4.98	746.42	30.66	841.65	34.57	846.53	34.80
2012	2811.90	5.20	843.20	29.99	1003.07	35.67	965.63	34.34
2013	3166.90	5.32	954.58	30.10	1139.38	36.00	1072.93	33.90
2014	3531.24	5.48	1057.92	29.96	1343.78	38.05	1129.54	31.99
2015	4097.46	5.95	1247.53	30.45	1650.67	40.29	1199.27	29.27
2016	4634.49	6.23	1391.03	30.01	1909.67	41.21	1333.79	28.78
2017	5259.83	6.36	1520.59	28.91	2225.88	42.32	1513.36	28.77
2018	5912.12	6.57	1639.91	27.74	2581.08	43.66	1691.20	28.61
2019	6584.14	6.64	1801.70	27.36	2915.06	44.27	1867.39	28.36
*r*	−0.839	−0.848	−0.834	−0.667	−0.796	−0.288	−0.895	0.516
*p*	< 0.01	< 0.01	< 0.01	< 0.01	< 0.01	0.122	< 0.01	< 0.01

*THE* total health expenditure; *GHE* government health expenditure; *OOPHE* out-of-pocket health expenditure; *SHE* social health expenditure; *GDP* gross domestic product

## Discussion

MMR is a universally accepted indicator to measure the status of a nation’s health and economic systems. This study assessed the trends of MMR, the main causes of maternal death, and the correlations between MMR and total health financing composition.

Although the gap in per capita annual income between the rural and urban areas widened from 1990 to 2019, the gap in MMR between them narrowed. This may be due to the improvement in medical services in rural areas [10]. Urbanization may be another reason for this phenomenon. With the emergence of urbanization, part of the rural population moved to cities. The economic status, health awareness, and educational background of this population are relatively low [29]. The MMRs in rural and urban areas were almost equal in 2010. This phenomenon may resulted from the implementation of the Basic Public Health Service Equalization project by Chinese government in 2009, which aimed to equalize public health services and improve the quality of life of all urban and rural residents [30]. The MMR in rural areas in China fluctuated between 1990 and 2002. After the implementation of the New Cooperative Medical Scheme in 2003, the MMR in rural areas steadily decreased from 2003 to 2019. The Chinese government carried out insurance and health care reform, resulting in more than 90% of the population having some medical insurance [31, 32]. These measures have likely helped to improve the maternal survival of Chinese women.

The mortalities caused by the main maternal diseases all declined from 1990 to 2019, which may be due to the improvement of healthcare conditions in China. The decline in mortality caused by obstetric hemorrhage seems to be an important contributor in the decline in maternal mortality in China. Prevention of obstetric hemorrhage is important for improving the survival rate of pregnant women. Measures to reduce the risk of obstetric hemorrhage include antenatal care, skilled delivery, emergency obstetric care, and postpartum care. These measures are more effective when combined with hospital delivery. Promoting hospital delivery is a very effective measure to reduce the risk of pregnancy-related diseases, especially in developing countries where many women traditionally give birth at home [33].

The proportion of OOPHE is positively correlated with underdevelopment and social injustice [18, 19]. Increasing the proportion of OOPHE may cause people to be unable to access care and may further compromise their financial security [34–36]. OOPHE always accounts for a large proportion of THE in developing countries [37]. Governmental health spending can effectively reduce the burden of OOPHE at the household level [38]. Although the proportion of OOPHE in THE decreased in China, China still has one of the highest OOPHE rates in Asia [18, 39]. The implementation of the zero-markup policy for essential drugs in China did lead to a decrease in cost of prescription drugs but has led to rising costs other hospital and health expenditure costs, so the actual OOPHE of patients is still very high [40]. Compared with developed countries, the proportion of GHE in THE in China was far less than the United States, Japan, Canada and Italy (all above 40%) [17]. In the case of limited GHE, SHE should be increased to alleviate the problem of “expensive medical service”. It is important to transfer individual medical burden and ensure the source of social funds as a safety net. Chinese government should expand the coverage of serious disease insurance, strengthen the construction of the medical security system, increase the reimbursement ratio, and include more safe and effective drugs in the national basic medical insurance drug list to reduce the burden of personal medical expenses.

In the past three decades, China has implemented various public health programs for maternal healthcare [41]. The Basic Public Health Service Equalization project was launched in 2009. This project aimed to equalize public health services and improve the quality of life of all urban and rural residents [30]. This project has numerous objectives, one of which is improving the quality of maternal health services. Specific measures included the establishment of maternal health records, prenatal examinations, and postnatal visits for rural and urban pregnant women. For example, maternal health providers were trained according to national standards and were required to deliver the same quality of maternal health services to rural and urban women [9]. The Maternal Mortality Reduction and Neonatal Tetanus Elimination program was implemented in 378 counties in 12 western provinces in 1999, expanding coverage to 2288 counties in 22 central and western provinces from 2008. This program effectively reduced the maternal mortality rate through the improvement of hospital delivery, and it was implemented at the national level to ensure free hospital delivery for all women in China in 2009 [10]. This program was composed of the following five strategies: pregnancy risk screening and assessment, case-by-case management, referral and treatment, reporting for maternal deaths, and accountability strategies [14]. The Urban Employees Basic Medical Insurance, Urban Residents Basic Medical Insurance, and New Cooperative Medical Scheme were health insurance programs funded by central and local governments and donations from individuals. The Urban Employees Basic Medical Insurance was a compulsory health insurance launched in 1999 for employees and employers in urban areas. However, older adults, children, students, and unemployed individuals who moved to urban areas from rural areas were not included in the Urban Employees Basic Medical Insurance system. Therefore, to address this problem, the Urban Residents Basic Medical Insurance scheme was established in 2007, and the scheme improved the healthcare services provided to some groups and reduced healthcare inequalities [11]. The New Cooperative Medical Scheme offered subsidies for rural residents working in antenatal and postnatal facilities and encouraged hospital delivery, either as a prepayment or retrospective reimbursement [42].

This study has some limitations. Data on maternal deaths in some extremely remote areas may not be registered. The quality of surveillance systems and data may differ across regions. Research has reported discrepancies between routine data and survey data on the number of reported live births and child and maternal mortality, and the quality of routine data in urban areas was better than in rural areas [43].

## Conclusions

China has made remarkable progress in improving maternal survival, especially in rural areas. The gap in MMR between rural and urban areas has narrowed substantially. To achieve the goal set by the Healthy China 2030 and further decrease the MMR, China should take effective measures to prevent obstetric hemorrhage, increase the quality of obstetric care, improve the efficiency and fairness of the government health funding, strengthen the medical security system, reduce income inequality, and allocate resources more rationally.

## Data Availability

The dataset generated and analyzed during the current study is available in the official website of National Health Commission of the People’s Republic of China. (http://www.nhc.gov.cn), and the public access to this dataset is open. The dataset is also available from the corresponding author on reasonable request.

## Abbreviations

MMR: Maternal mortality ratio
UN: United Nation
GDP: Gross domestic product
NMMSS: National maternal mortality surveillance system
THE: Total health expenditure
GHE: Government health expenditure
OOPHE: Out-of-pocket health expenditure
SHE: Social health expenditure
